# Patients report high information coordination between rostered primary care physicians and specialists: A cross-sectional study

**DOI:** 10.1371/journal.pone.0307611

**Published:** 2024-08-22

**Authors:** Bahram Rahman, Glenda Babe, Lauren E. Griffith, David Price, Lauren Lapointe-Shaw, Andrew P. Costa

**Affiliations:** 1 Physician and Provider Services Division, Ministry of Health, Toronto, Ontario, Canada; 2 Department of Health Research Methods, Evidence, and Impact, McMaster University, Hamilton, Ontario, Canada; 3 ICES (formerly known as the Institute for Clinical Evaluative Sciences), Toronto, Ontario, Canada; 4 McMaster Family Health Team, Hamilton, Ontario, Canada; 5 Medical School, Simon Fraser University, Burnaby, British Columbia, Canada; 6 Institute of Health Policy, Management and Evaluation, University of Toronto, Toronto, Ontario, Canada; 7 Division of General Internal Medicine and Geriatrics, University Health Network and Sinai Health System, Toronto, Ontario, Canada; 8 Department of Medicine, University of Toronto, Toronto, Ontario, Canada; 9 Women’s College Institute for Health System Solutions and Virtual Care, Women’s College Hospital, Toronto, Ontario, Canada; 10 Centre for Health Economics and Policy Analysis, McMaster University, Hamilton, Ontario, Canada; 11 Department of Medicine, McMaster University, Hamilton, Ontario, Canada; 12 The Research Institute of St. Joe’s Hamilton, St. Joseph’s Healthcare Hamilton, Hamilton, Ontario, Canada; 13 Centre for Integrated Care, St. Joseph’s Health System, Hamilton, Ontario, Canada; 14 Schlegel Research Institute for Aging, Waterloo, Ontario, Canada; Università degli Studi di Milano: Universita degli Studi di Milano, ITALY

## Abstract

Our study aimed to describe patient experience of information coordination between their primary care physician and specialists and to examine the associations between their experience and their personal and primary care characteristics. We conducted a cross-sectional study of Ontario residents rostered to a primary care physician and visited a specialist physician in the previous 12 months by linking population-based health administrative data to the Health Care Experience Survey collected between 2013 and 2020. We described respondents’ sociodemographic and health care utilization characteristics and their experience of information coordination between their primary care physician and specialists. We measured the adjusted association between patient-reported measures of information coordination before and after respondents received care from a specialist physician and their type of primary care model. 1,460 out 20,422 (weighted 7.5%) of the respondents reported that their specialist physician did not have basic medical information about their visit from their primary care physician in the previous 12 months. 2,298 out of 16,442 (weighted 14.9%) of the respondents reported that their primary care physician seemed uninformed about the care they received from the specialist. Females, younger individuals, those with a college or undergraduate level of education, and users of walk-in clinics had a higher likelihood of reporting a lack of information coordination between the primary care and specialist physicians. Only respondents rostered to an enhanced fee-for-service model had a higher odds of reporting that the specialist physician did not have basic medical information about their visit compared to those rostered to a Family Health Team (OR 1.22, 95% Cl 1.12–1.40). We found no significant association between respondent’s type of primary care model and that their primary care physician was uninformed about the care received from the specialist physician. In this population-based health study, respondents reported high information coordination between their primary care physician and specialists. Except for respondents rostered to an enhanced fee-for-service model of care, we did not find any difference in information coordination across other primary care models.

## Introduction

Primary care is the first point of contact with the health care system and often coordinates services with other parts of the health care system, including care provided by specialist physicians [1, 2]. Information coordination is defined as the degree which patient medical information from different sources is shared and incorporated into patient’s current health care plan [3–5]. In Canada, health care is publicly funded through federal and provincial taxation with the federal government transferring funds to provinces and territories, which are responsible for administering and delivering health care services, including physician services [2]. In Ontario, patients typically need a referral from a physician or nurse practitioner to see a specialist physician. The gatekeeping role of primary care is reinforced through guidelines relevant to the practice of medicine and financial incentives [6, 7]. Recent reports suggest that 27% Ontario’s population (3.9 million) had a consultation with a specialist physician referred by a primary care physician [8]. Primary care physicians are required to communicate the medical reason for the patient’s visit to the specialist [7]. Once the course of care is completed, the specialist must transmit details of their findings and recommendations back to the primary care physician [7, 9].

Gaps in patient care information could undermine the quality-of-care patients receive and their satisfaction with care, particularly for complex patients who might feel overwhelmed navigating the health care system [10–12]. A longitudinal relationship to a primary care physician has shown to improve coordination of care with specialists [13, 14]. Also, information coordination with specialists have been shown to improve a patient experience, patient trust, chronic disease management (i.e., diabetes, cancer, COPD) and management of mental health issues [11, 15–17]. A physician survey showed that although 98% of primary care physicians sent information to specialists, only 67% received information back from the specialist physician about changes made to their patient’s medication or health care plan [18]. It is also reported that a greater use of specialist services may reduce information coordination among physicians [19–23].

In Ontario, different primary care models were introduced though a series of reform since early 2000 [24, 25]. These models are highly diverse in terms of their characteristics, governance and financial incentives to provide services for patients. While some models are paid per capita or salaried receive and receive support from a team of allied health care professionals including social works and care coordinators, such support is not available for 70% of Ontarians [24–26].

Patients directly experience the impact of information coordination in their care, influencing their perception of their overall health and their behavior of accessing health care services [27, 28]. Existing evidence on information coordination between primary care and specialist physicians has mostly focused on small patient populations [29–31], specific health conditions (e.g., depression, diabetes, cancer) [16, 17, 32], and reported the experiences of providers in coordinating information [10, 19, 33]. Also, large scale and multiyear data on patients’ experience of information coordination between primary care and specialist physicians are rarely available for research studies. Such data are necessary to understand the quality of information coordination between primary care and specialist physicians as experienced by patients.

Our objective was to describe patient reported experience on information coordination between their rostered primary care physician and specialist physician. We also wanted to examine the associations between their experience of information coordination and their personal and primary care characteristics. We hypothesized that respondents’ primary care model would be associated with the experience of information coordination between their rostered primary care physician and specialist physician.

## Methods

### Study design and setting

We conducted a cross-sectional study of Ontario residents who had a primary care physician and reported a visit to a specialist in the previous 12 months. We linked population-level administrative data to Ontario’s Health Care Experience Survey (HCES) collected between 2013 and 2020 using unique encoded identifiers.

Ontario had over 15.4 million residents in January 2023 and is the most populous province in Canada [34]. Physician services are publicly funded in Ontario and in most cases, delivered by private physician practices or non-for-profit organizations through different payment models. Over 87% of the population is formally rostered with a primary care physician in a patient enrolment model [24]. The remaining population receives care from fee-for-service or salaried primary care physicians nurse-practitioner-led clinics or do not have a primary care physician [24]. Patient enrolment models combine formal patient registration, blended funding consisting of a varying proportion of capitation payments, and bonuses for meeting preventive care targets [24]. Specialists are paid through a combination of alternative funding agreements and fee-for-service and can practice solo or as a team in the community or in a hospital setting [35]. The majority (87%) of physicians use an electronic medical record (EMR) system [36]. Yet, physicians still commonly use fax machines to send patient information [36] and only 37% of the primary care physicians use electronic referral system to exchange clinical information with a specialist physician outside their practice [36]. The use of data in this study was authorized under section 45 of *Ontario’s Personal Health Information Protection Act*, *2004* and did not require ethics review board approval.

### Data sources

We linked the HCES survey data using unique anonymized identifiers with other health administrative datasets (S1 Appendix lists databases used) housed at ICES (formerly known as Institute for Clinical Evaluative Sciences) in September 2021. ICES is an independent, non-profit research institute whose legal status under Ontario’s health information privacy law allows it to collect and analyze health care and demographic data, without consent, for health system evaluation and improvement.

The HCES is a large population based cross-sectional household survey of Ontario residents created by the Ministries of Health and Long-Term Care to report on patients’ experience with the primary care system [37]. It has been active from 2012 to the present and is administered by telephone (mobile phone or landline) in English or French, to a target population that is 16 years and older living in private dwellings.

Potential participants for the HCES are selected from the Registered Persons Database (RPDB) which contains information on persons registered under the universal Ontario Health Insurance Plan (OHIP). The sample is collected using a stratified design (Ontario is first divided into 76 population strata, households were randomly selected from each stratum, and then one respondent is randomly selected from each household) [37]. Once a household is sampled, they are removed from the sampling frame for two years [38]. The survey excluded people living in institutions and households without telephones [37]. The total sample size of the survey was 11,200 each year, from 2013 to 2020 [37]. The overall response rate was 47.3% for our study period and ranged from 29% to 54% in each survey year [37].

### Study population

Our study population included all participants of the HCES from Wave 2, January 2013 (the first complete survey wave after the pilot) to Wave 29, February 2020 (the last survey wave before pausing due to COVID-19) who reported seeing a specialist in past 12 months. We excluded those who were under 16 years old, reported not having a primary care provider and those who could not be linked to a primary care physician in the OHIP system on April 1 of the interview year.

**Fig 1:**
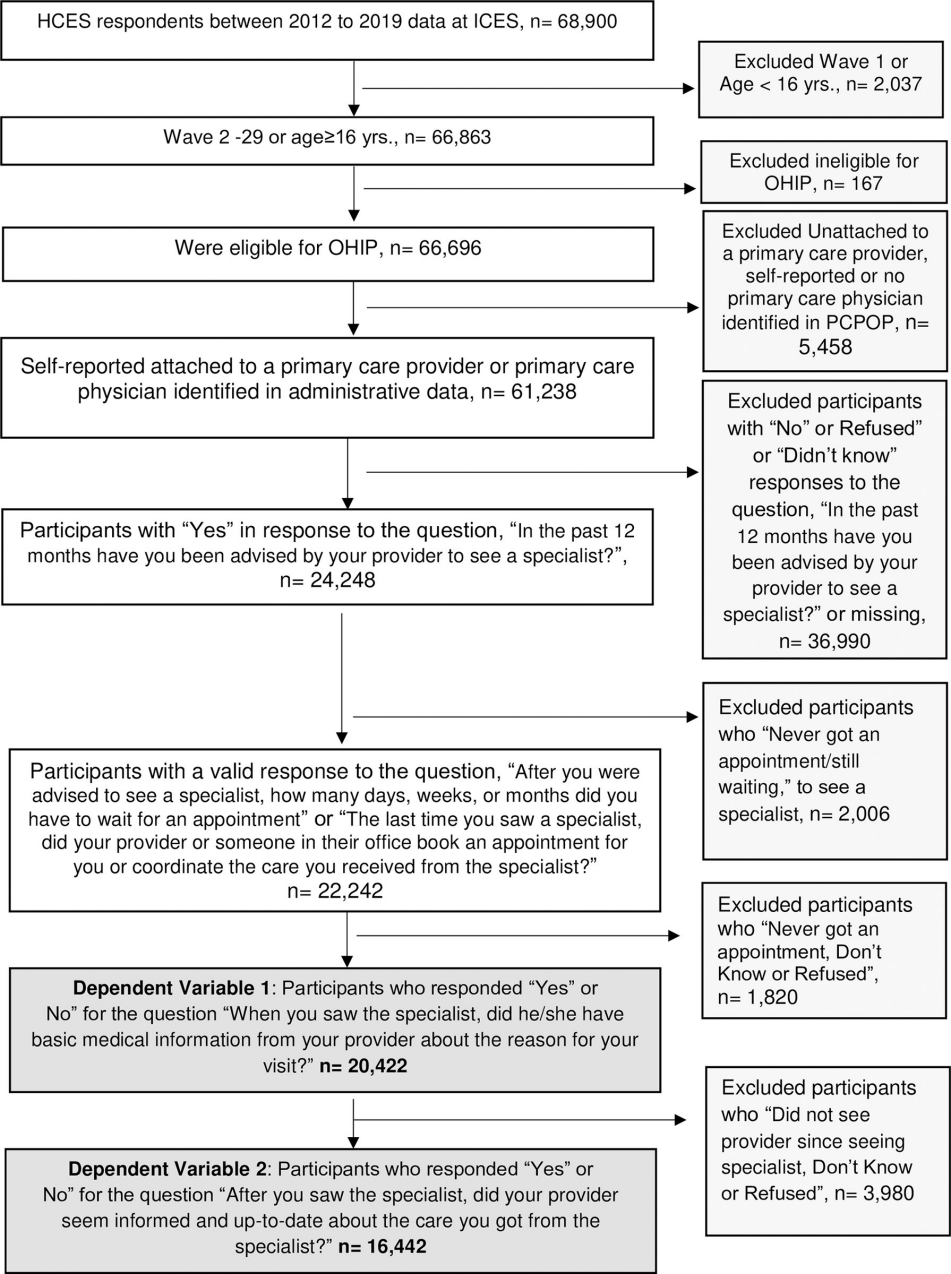
Inclusion and exclusion criteria for study population.

To analyze information coordination from the primary care physician to the specialist, we restricted the study population to those who reported “Yes” or “No” to the question: “When you last saw the specialist, did he/she have basic medical information from your [primary care] provider about the reason for your visit?”. And to analyze information coordination back to primary care from the specialist, we restricted the study population to those who reported “Yes” or “No” to the question: “After you saw the specialist, did your [primary care] provider seem informed and up to date about the care you got from the specialist?”. We excluded those who responded, “don’t know,” “refused” or were missing in each question. Fig 1 presents the complete exclusion criteria of the study population.

### Dependent measures

Our two dependent measures were based on the survey questions: “When you last saw the specialist, did he/she have basic medical information from your [primary care] provider about the reason for your visit?” or “After you saw the specialist, did your [primary care] provider seem informed and up to date about the care you got from the specialist?” A “No” response was coded as 0 and a “Yes” response was coded as 1 in each question.

### Independent measures

We included respondents’ age, sex, self-reported level of education, self-reported financial situation, the most commonly spoken language at home, self-reported wait-time to see the referred specialist, and self-reported use of walk-in clinic in the previous 12 months. Walk-in clinics provide medical services without requiring an appointment or referral for people who do not have a primary care physician or have one but are unable to reach them [38].

Patient’s rurality was calculated using the Rurality Index of Ontario (Large urban = 0; Medium urban = 1–9; Small urban = 10–39; and Rural ≥ 40). We determined respondent’s general medical complexity score using the CIHI Grouping Methodology at the time of the interview [39]. Respondents’ primary care model and rostered primary care physician, (including formally enrolled and virtually rostered to the physician with the highest total value of fee-for-service billing claims for primary care services over the previous 2 years [40] were obtained from the OHIP billing system. The total number of visits to the rostered primary care physician, the total number of visits to any specialists, and the number of specialties visited were calculated using physician billing data over the previous two years from the interview date. For descriptive purposes only, we included the total number of visits to any primary care physician, whether the patient reported receiving conflicting information from their rostered primary care physician and a specialist physician (data available only between wave 20 and 27 of the survey), whether the primary care physician or someone else in the office helped to book the specialist appointment or coordinated the care with the specialist physician (available only between wave 6 and 15). S2 Appendix includes the complete description of variables and data sources.

### Statistical analysis

We reported demographic characteristics, count of visits to rostered primary care physician, count of visits to a specialist and respondent’s complexity score using weighted mean and standard deviation. For categorical variables, we reported raw counts and weighted proportions. We also calculated standardized mean difference, with difference greater than 10% considered meaningful [41]. We used separate multivariable logistic regression models to estimate unadjusted and adjusted odds ratios for each of the dependent variables (complete-case analysis). Data imputation was deemed unnecessary as the proportion of missing data was smaller than 5% [42]. We used generalized estimating equations with an exchangeable correlation structure to account for clustering at the level of rostered physician. We adjusted for age, sex, most commonly spoken language at home, self-reported levels of education, self-reported financial situation, self-reported wait-time to see a specialist, use of walk-in clinics in the previous 12 months, respondent’s primary care model, complexity score, total number of specialist visits, total number of visits to rostered primary care physician, and count of unique specialties visited over two years. We reported the results as adjusted odds ratios (ORs) with 95% confidence intervals (CIs).

We weighted all results using the post-stratification weight calculated from respondents’ age, sex, survey strata, total population in each stratum and the sampling probability weight. We considered a 2-tailed *p* value of less than 0.05 significant. We conducted all analysis using SAS Enterprise Guide software, Version 8.3 Copyright© 2020 SAS Institute Inc.

## Results

After exclusions (Fig 1), there were 20,422 participants providing yes/no responses for our first dependent measure (whether specialist had basic medical information about reason for the visit from the primary care physician) and 16,442 for our second dependent measure (primary care physician seemed uninformed about the care received from the specialist physician). Table 1 presents the sample characteristics for both groups.

**Table 1:** Baseline characteristic of each respondent groups, Ontario Health Experience Survey, waves 2 to 29 (January 2013—February 2020).

Respondents’ Characteristics	Specialist had basic medical information from the primary care physician about the reason for the visit	Primary care physician seemed informed about the care received from the specialist physician
	(n = 20,422)	(n = 16,442)
	n (%)	n (%)
**Sex**		
Female	12,275 (55.2)	9,807 (55.2)
Male	8,147 (44.8)	6,635 (44.8)
**Age** (mean, SE)	51.3 (0.19)	51.5 (0.21)
**Age category**		
16–39		
40–64	9,695 (42.7)	7,727 (42.2)
65–84	6,182 (26.5)	5,094 (27,1)
85+	472 (1.7)	401 (1.8)
**Rurality**		
Large urban (RIO score 0)	7,352 (43.0)	6,004 (43.5)
Medium urban (RIO score 1–9)	5,447 (27.8)	4,424 (28.1)
Small urban (RIO score 10–39)	5,139 (20.6)	4,073 (20.1)
Rural (RIO score 40+)	2,484 (8.6)	1,941 (8.3)
**Self-reported education**		
High school	5,959 (27.0)	4,936 (27.8)
College or bachelor’s degree	11,712 (58.3)	9,306 (57.4)
Graduate or professional degree	2,589 (14.0)	2,068 (14.0)
Missing	162 (0.7)	132 (0.8)
**Self-reported financial situation**		
Very comfortable	3,229 (15.8)	2,554 (15.6)
Comfortable	11,987 (59.6)	9,662 (59.8)
Tight/very tight/poor	4,663 (22.0)	3,799 (22.1)
Don’t know or refused	543 (2.6)	427 (2.4)
**Language most often spoken at home**		
English or French	18,537 (86.8)	14,826 (86.0)
Other than English or French	1,885 (13.2)	1,616 (14.0)
**Primary care physician or clinic booked the appointment or coordinated care with the specialist** *		
Yes	5,378 (79.7)	4,306 (80.4)
No	1,245 (19.0)	925 (18.3)
NA/I don’t know	95 (1.3)	72 (1.3)
**Received conflicting information from primary care provider and specialist** **		
Yes	938 (13.0)	807 (13.6)
No	6,001 (83.2)	4,995 (84.2)
Don’t know/Refused	295 (3.8)	134 (2.2)
**Self-reported time waited to see a specialist**		
2 weeks	5,751 (29.1)	4,685 (29.8)
3–8 weeks	8,411 (41.0)	6,779 (40.9)
More than 8 weeks	6,260 (29.9)	4,978 (29.3)
**Number of specialty types receiving care from**		
1 type	2,216 (13.1)	1,719 (12.6)
2 types	2,980 (15.9)	2,248 (14.7)
3 types or more	15,226 (71.0)	12,475 (72.8)
**Types of primary care models** ***		
Solo FFS	861 (4.8)	708 (5.0)
Enhanced FFS	5,647 (32.4)	4,728 (33.4)
Non-team Capitation	6,231 (32.1)	4,948 (31.6)
Team Capitation	7,100 (29.9)	5,611 (29.2)
Other PEM models	583 (0.9)	447 (0.8)
**Self-reported use of a walk-in clinic in the last 12 months**		
Yes	6,110 (33.7)	5,025 (34.1)
No	14,201 (65.8)	11,330 (65.4)
I don’t know/Refused	111 (0.5)	87 (0.5)
**Complexity score based on CIHI Pop Grouper** (mean, SE)	1.6 (0.03)	1.7 (0.03)
**Total visits to the rostered primary care physician over two years** (mean, SE)	8.9 (0.12)	9.4 (0.14)
**Total visits to any specialist physicians over two years** (mean, SE)	15.0 (0.15)	15.8 (0.17)

Note: Reporting raw counts, weighted proportions, and weighted means.

*Added in wave 6 and dropped in wave 15 of the survey and calculated for smaller sample size.

** Added in wave 20 of the survey and calculated for smaller sample size.

*** Solo FFS: Patients are not formally part of an enrolment model but receive care from a regular primary care physician who is paid purely fee-for-service. Enhanced Fee-for-Service includes Comprehensive Care Model and Family Health Group where physicians are paid a mix of fee-for-service along with bonuses and premiums. Non-team Capitation includes Capitation models, i.e., Family Health Organization and Family Health Network where physicians are paid a mix of capitation payment, bonuses, premiums, and fee-for-service but they are not signatory to a Family Health Team (FHT). FHTs are interdisciplinary models of care. Team Capitation: Capitation models, i.e., Family Health Organization and Family Health Network, are part of a Family Health Team (FHT). Other PEM models include smaller specialized patient enrolment models.

### Descriptive comparisons

7.5% (n = 1,460) of the respondents reported that in the previous 12 months their specialist physician did not have basic medical information from the primary care physician about the reason for the visit. These respondents were more likely to be female, younger and a greater proportion of them reported having college or undergraduate levels of education. A higher proportion of them also reported that their primary care physician or clinic did not book or coordinate their care with the specialist physician (46.5% vs. 16.4%, SMD = 0.68) and received conflicting information from their primary care physician and specialist physician (23.0% vs. 12.2%, SMD = 0.28). They also visited a walk-in clinic in the last 12 months (43.9% vs. 32.8%, SMD = 0.23) were less complex (mean of complexity score 1.4 vs. 1.7, SMD = 0.10) and had a lower total number of visits to any specialist physician over the 2 years period (mean 13.4 vs. 15.1, SMD = 0.10) (Table 2).

**Table 2:** Descriptive comparison of those who reported that the specialist had basic medical information from the primary care physician about the reason for the visit (n = 20,422).

Respondents’ Characteristics	Specialist had basic medical information from the primary care physician about the reason for the visit n (%)		Standardized Mean Difference
	Yes	No	
	18,962 (92.5)	1,460 (7.5)	
**Sex**			
Female	11,324 (54.8)	951 (60.1)	0.11
Male	7.638 (45.2)	509 (39.9)	
**Age** (mean, SE)	51.7 (0.19)	46.0 (0.57)	0.32
**Age category**			
16–39	3,639 (28.2)	434 (40.6)	0.26
40–64	9,006 (42.8)	689 (41.4)	-0.03
65–84	5,869 (27.3)	313 (17.0)	-0.25
85+	448 (1.7)	24 (1.0)	-0.06
**Rurality**			
Large urban (RIO score 0)	6,779 (42.7)	573 (47.0)	0.09
Medium urban (RIO score 1–9)	5,047 (27.8)	400 (27.9)	0.00
Small urban (RIO score 10–39)	4,800 (20.8)	339 (18.3)	-0.06
Rural (RIO score 40+)	2,336 (8.7)	148 (6.8)	-0.07
**Self-reported education**			
High school	5.614 (27.5)	345 (20.8)	-0.16
College or bachelor’s degree	10,799 (57.7)	913 (64.7)	0.14
Graduate-professional degree	2,394 (14.1)	195 (14.1)	0.00
Missing	155 (0.7)	7 (0.4)	
**Self-reported financial situation**			
Very comfortable	3,010 (15.8)	219 (15.5)	0.00
Comfortable	11,164 (59.8)	823 (57.3)	-0.05
Tight/very tight/poor	4,289 (21.8)	374 (24.3)	0.06
Don’t know or refused	499 (2.6)	44 (2.9)	0.03
**Language most often spoken at home**			
English or French	17,211 (86.7)	1,326 (87.9)	
Other	1,751 (13.3)	134 (12.1)	0.04
**Primary care physician or clinic booked the appointment or coordinated care with the specialist** *			
Yes	5,099 (82.2)	279 (52.1)	-0.67
No	1,013 (16.4)	232 (46.5)	0.68
NA/I don’t know	86 (13.4)	9 (1.4)	0.00
**Received conflicting information from primary care provider and specialist** **			
Yes	823 (12.2)	115 (23.0)	0.28
No	5,666 (84.1)	335 (71.6)	-0.30
Don’t know/Refused	263 (3.7)	32 (5.4)	0.08
**Self-reported waited to see a specialist**			
2 weeks	5,327 (29.1)	424 (29.3)	0.00
3–8 weeks	7,891(41.3)	520 (37.7)	-0.07
More than 8 weeks	5,744 (29.6)	516 (33.1)	0.07
**Number of specialty types receiving care from**			
1 type	1,977 (12.6)	239 (19.7)	0.20
2 types	2,756 (15.8)	224 (16.4)	0.02
3 types or more	14,229 (71.6)	997 (63.9)	-0.17
**Types of primary care models** ***			
Solo FFS	795 (4.7)	66 (5.3)	0.01
Enhanced FFS	5,185 (32.1)	462 (35.7)	0.03
Non-team Capitation	5,792 (32.1)	439 (32.4)	-0.09
Team Capitation	6,637 (30.2)	463 (26.0)	-0.04
Other PEM models	553 (0.9)	30 (0.6)	0.08
**Self-reported use of a walk-in clinic in the last 12 months**			
Yes	5,532 (32.8)	578 (43.9)	0.23
No	13,331 (66.7)	870 (55.0)	-0.24
I don’t know/Refused	99 (0.5)	12 (1.1)	0.07
**Complexity score based on CIHI Pop Grouper (**mean, SE)	1.7 (0.03)	1.4 (0.06)	0.10
**Total visits to any primary care physician over two years** (mean, SE)	13.0 (0.14)	12.6 (0.45)	0.03
**Total visits to the rostered primary care physician over two years** (mean, SE)	8.9 (0.12)	8.1 (0.30)	0.09
**Total visits to any specialist physicians over two years** (mean, SE)	15.1 (0.16)	13.4 (0.48)	0.10

Note: Reporting raw counts, weighted proportions, and weighted means.

*Added in wave 6 and dropped in wave 15 of the survey and calculated for smaller sample size.

** Added in wave 20 of the survey and calculated for smaller sample size.

***Solo FFS: Patients are not formally part of an enrolment model but receive care from a regular primary care physician who is paid purely fee-for-service. Enhanced Fee-for-Service includes Comprehensive Care Model and Family Health Group where physicians are paid a mix of fee-for-service along with bonuses and premiums. Non-team Capitation includes Capitation models, i.e., Family Health Organization and Family Health Network where physicians are paid a mix of capitation payment, bonuses, premiums, and fee-for-service but they are not signatory to a Family Health Team (FHT). FHTs are interdisciplinary models of care. Team Capitation: Capitation models, i.e., Family Health Organization and Family Health Network, are part of a Family Health Team (FHT). Other PEM models include smaller specialized patient enrolment models.

14.9% (n = 2,298) of the respondents reported that their primary care physician seemed uninformed about the care they received from the specialist physician. These respondents were also more likely to be female, younger and a greater proportion of them had a college or undergraduate level of education. A higher proportion of them received conflicting information from primary care physician and specialist physician (22.3% vs. 12.2%, SMD = 0.45). They were more likely to visit only 1 type of specialist over 2 years period (17.7% vs. 11.7%, SMD = 0.18) and a higher proportion of them reported visited a walk-in clinic in the previous 12 months (45.1% vs. 32.2%, SMD = 0.27). They were less likely to be complex (mean of complexity score 1.2 vs. 1.8, SMD = 0.28), had lower number of visits to their primary care physician (7.7 visits vs. 9.7 visits, SMD = 0.22) and lower number of visits to any specialist physician over the 2 years period (mean 12.7 vs. 16.3, SMD = 0.23) (Table 3).

**Table 3:** Descriptive analysis of those who reported that their primary care physician seemed uninformed about the care received from the specialist physician (n = 16,442).

Respondents’ Characteristics	Primary care physician seemed informed about the care received from the specialist physician n (%)		Standardized Mean Difference
	Yes	No	
	14,144 (85.1)	2,298 (14.9)	
**Sex**			
Female	8,332 (54.3)	1,457 (60.4)	0.13
Male	5,912 (45.7)	823 (39.6)	
**Age** (mean, SE)	52.9 (0.22)	44.0 (0.42)	0.51
**Age category**			
16–39	2,433 (25.1)	787 (45.5)	0.42
40–64	6,618 (42.4)	1,109 (40.8)	-0.03
65–84	4,715 (29.6)	379 (12.9)	-0.42
85+	373 (1.9)	23 (0.8)	-0.1
**Rurality**			
Large urban (RIO score 0)	5,165 (43.4)	839 (44.0)	0.01
Medium urban (RIO score 1–9)	3,756 (27.8)	668 (29.6)	0.04
Small urban (RIO score 10–39)	3,530 (20.2)	543 (19.6)	-0.02
Rural (RIO score 40+)	1,693 (8.6)	248 (6.8)	-0.07
**Self-reported education**			
High school	4,428 (29.2)	508 (20.2)	-0.21
College or bachelor’s degree	7,864 (56.1)	1,442 (64.6)	0.18
Graduate-professional degree	1,734 (13.9)	334 (14.4)	0.02
Missing	118 (0.8)	14 (0.8)	
**Self-reported financial situation**			
Very comfortable	2,215 (15.8)	339 (14.1)	-0.05
Comfortable	8,371 (60.3)	1,291 (56.9)	-0.07
Tight/very tight/poor	3,177 (31.3)	622 (26.9)	-0.13
Don’t know or refused	381 (2.6)	46 (2.1)	-0.03
**Language most often spoken at home**			
English or French	12,784 (86.2)	2,042 (85.0)	-0.03
Other	1,360 (13.8)	256 (15.0)	
**Primary care physician or clinic booked the appointment or coordinated care with the specialist** *			
Yes	3,780 (83.3)	526 (64.5)	0.27
No	677 (15.4)	248 (34.4)	-0.30
NA/I don’t know	63 (1.3)	9 (1.1)	0.11
**Received conflicting information from primary care provider and specialist** **			
Yes	4,440 (85.9)	555 (73.9)	-0.44
No	613 (12.2)	194 (22.3)	0.45
Don’t know/Refused	103 (1.9)	31 (3.8)	-0.02
**Self-reported waited to see a specialist**			
2 weeks	4,184 (30.8)	501 (24.2)	-0.15
3–8 weeks	5,895 (41.2)	884 (39.6)	-0.03
More than 8 weeks	4,065 (28.0)	913 (36.2)	0.18
**Number of specialty types receiving care from**			
1 type	1,379 (11.7)	340 (17.7)	0.18
2 types	1,864 (14.1)	384 (17.9)	0.10
3 types or more	10,901 (74.2)	1,574 (64.4)	-0.22
**Types of primary care models** ***			
Solo FFS	608 (4.9)	100 (5.6)	-0.03
Enhanced FFS	4,023 (33.2)	705 (34.7)	0.03
Non-team Capitation	4,283 (31.7)	665 (30.3)	-0.02
Team Capitation	4,843 (29.3)	768 (28.6)	-0.01
Other PEM models	387 (0.9)	60 (0.8)	0.03
**Self-reported use of a walk-in clinic in the last 12 months**			
Yes	4,090 (32.2)	935 (45.1)	0.27
No	9,983 (67.3)	1,347 (54.1)	-0.27
I don’t know/Refused	71 (0.5)	16 (0.8)	0.04
**Complexity score based on CIHI Pop Grouper** (mean, SE)	1.8 (0.03)	1.2 (0.04)	0.28
**Total visits to any primary care physician over two years** (mean, SE)	13.9 (0.17)	12.0 (0.34)	0.15
**Total visits to the rostered primary care physician over two years** (mean, SE)	9.7 (0.16)	7.7 (0.20)	0.22
**Total visits to any specialist physicians over two years** (mean, SE)	16.3 (0.18)	12.7 (0.34)	0.23

Note: Reporting raw counts, weighted proportions, and weighted means.

*Added in wave 6 and dropped in wave 15 of the survey and calculated for smaller sample size. Included only for descriptive purposes.

** Added in wave 20 of the survey and calculated for smaller sample size. Included only for descriptive purposes.

*** Solo FFS: Patients are not formally part of an enrolment model but receive care from a regular primary care physician who is paid purely fee-for-service. Enhanced Fee-for-Service includes Comprehensive Care Model and Family Health Group where physicians are paid a mix of fee-for-service along with bonuses and premiums. Non-team Capitation includes Capitation models, i.e., Family Health Organization and Family Health Network where physicians are paid a mix of capitation payment, bonuses, premiums, and fee-for-service but they are not signatory to a Family Health Team (FHT). FHTs are interdisciplinary models of care. Team Capitation: Capitation models, i.e., Family Health Organization and Family Health Network, are part of a Family Health Team (FHT). Other PEM models include smaller specialized patient enrolment models.

### Adjusted association

Specialist did not have basic medical information from the primary care physician about the reason for the visit.

Female respondents and younger respondents had a higher odds reporting that their specialised physician did not have basic medical information from the primary care physician about the reason for the visit compared to male and older respondents. Respondents with a walk-in clinic visit in the previous 12 months also had a higher odds of reporting that the specialist physician did not have basic medical information from the primary care physician about the reason for the visit comparing to non-walk-in users (aOR 1.39, 95% Cl 1.19–1.61). Those who visited to more than one type of specialist had a lower odds of reporting that the specialist physician was uninformed about the reason for their visits compared to those visiting only 1 type of specialist. Only respondents rostered to an enhanced fee-for-service model (Family Health Group and Comprehensive Care Model) had a higher odds of reporting that the specialist physician did not have basic medical information from the primary care physician about the reason for the visit comparing to those rostered to a Family Health Team (aOR 1.22, 95% Cl 1.12–1.40) (Table 4).

**Table 4:** Adjusted odds ratios for dependent variables.

Respondents’ Characteristics	Specialist did not have basic medical information from the primary care physician about the reason for the visit		Primary care physician seemed uninformed about the care received from the specialist physician	
	(n = 19,679 –missing 743)		(n = 15,827 –missing 615)	
	Unadjusted OR (CL)	Adjusted OR (CL)	Unadjusted OR (CL)	Adjusted OR (CL)
**Sex**				
Male	Ref	Ref	Ref	Ref
Female	1.25 (1.09–1.43)	1.20 (1.14–1.38)	1.28 (1.14–1.43)	1.17 (1.04–1.31)
**Age category**				
16–39 (ref)	Ref	Ref	Ref	Ref
40–64	0.67 (0.58–0.78)	0.73 (0.62–0.87)	0.55 (0.48–0.62)	0.58 (0.51–0.66)
65–84	0.43 (0.36–0.52)	0.55 (0.44–0.68)	0.25 (0.21–0.29)	0.35 (0.29–0.42)
85+	0.40 (0.24–0.65)	0.57 (0.34–0.94)	0.24 (0.15–0.40)	0.44 (0.27–0.73)
**Self-reported education**				
Graduate or professional degree	Ref	Ref	Ref	Ref
High school	0.76 (0.61–0.95)	0.80 (0.63–1.02)	0.67 (0.55–0.81)	0.72 (0.59–0.88)
College or bachelor’s degree	1.12 (0.92–1.36)	1.06 (0.86–1.29)	1.11 (0.95–1.30)	1.02 (0.86–1.20)
**Self-reported financial situation**				
Very comfortable	Ref	Ref	Ref	Ref
Comfortable	1.14 (0.92–1.42)	1.05 (0.83–1.32)	1.41 (1.17–1.69)	1.26 (1.05–1.53)
Tight/very tight/poor	0.97 (0.81–1.18)	0.93 (0.77–1.14)	1.06 (0.89–1.24)	0.98 (0.83–1.14)
Don’t know or refused	1.16 (0.78–1.74)	1.29 (0.85–1.96)	0.89 (0.58–1.37)	0.84 (0.54–1.30)
**Language most often spoken at home**				
English or French	Ref	Ref	Ref	Ref
Other than English or French	1.89 (1.72–2.12)	0.76 (0.60–0.96)	1.10 (1.01–1.28)	0.90 (0.76–1.08)
**Self-reported waited to see a specialist**				
2 weeks	Ref	Ref	Ref	Ref
3–8 weeks	0.91 (0.77–1.07)	0.95 (0.80–1.13)	1.23 (1.06–1.42)	1.2 (1.05–1.41)
More than 8 weeks	1.11 (0.93–1.31)	1.17 (0.98–1.39)	1.64 (1.41–1.91)	1.63 (1.40–1.80)
**Number of specialty types receiving care from**				
1 type	Ref	Ref	Ref	Ref
2 types	0.66 (0.52–0.84)	0.72 (0.55–0.95)	0.83 (0.68–1.01)	0.93 (0.75–1.17)
3 types or more	0.57 (0.48–0.68)	0.71 (0.6.0–0.96)	0.57 (0.48–0.67)	0.89 (0.72–1.10)
**Types of primary care models^**				
Team Capitation	Ref	Ref	Ref	Ref
Solo FFS	1.29 (0.92–1.80)	1.29 (0.91–1.80)	1.17 (0.87–1.57)	1.04 (0.77–1.14)
Enhanced FFS	1.28 (1.09–1.52)	1.22 (1.12–1.40)	1.07 (0.93–1.23)	0.93 (0.79–1.08)
Non-team Capitation	1.17 (1.01–1.38)	1.11 (0.93–1.32)	0.98 (0.85–1.13)	0.91 (0.78–1.05)
Other PEM models	0.75 (0.36–1.57)	0.82 (0.38–1.07)	0.94 (0.59–1.38)	1.05 (0.67–1.64)
**Self-reported use of a walk-in clinic in the last 12 months**				
No	Ref	Ref	Ref	Ref
Yes	1.62 (1.41–1.86)	1.39 (1.19–1.61)	1.74 (1.55–1.95)	1.40 (1.24–1.58)
I don’t know/Refused	2.95 (1.46–5.96)	3.01 (1.48–6.30)	2.17 (0.12–1.43)	0.44 (0.18–1.10)
**Complexity score based on CIHI Pop Grouper** (1 unit increase)	0.95 (0.92–0.98)	1.01 (0.98–1.04)	0.85 (0.82–0.88)	0.91 (0.90–0.97)
**Total visits to the rostered primary care physician over two years** (1 visit increase)	0.98 (0.97–0.99)	0.99 (0.98–1.01)	0.97 (0.95–0.98)	0.98 (0.97–0.99)
**Total visits to any specialist physicians over two years** (1 visit increase)	0.99 (0.98–0.99)	1.00 (0.99–1.00)	0.98 (0.97–0.99)	0.99 (0.99–1.01)

Note: Rurality was excluded from the final multivariable regression model as it remained statistically not significant in both unadjusted and adjusted models. RIO’s unadjusted are included in S3 Appendix.

^ Solo FFS: Patients are not formally part of an enrolment model but receive care from a regular primary care physician who is paid purely fee-for-service. Enhanced Fee-for-Service includes Comprehensive Care Model and Family Health Group where physicians are paid a mix of fee-for-service along with bonuses and premiums. Non-team Capitation includes Capitation models, i.e., Family Health Organization and Family Health Network where physicians are paid a mix of capitation payment, bonuses, premiums, and fee-for-service but they are not signatory to a Family Health Team (FHT). FHTs are interdisciplinary models of care. Team Capitation: Capitation models, i.e., Family Health Organization and Family Health Network, are part of a Family Health Team (FHT). Other PEM models include smaller specialized patient enrolment models.

### Adjusted associations

Primary care physician seemed uninformed about the care received from the specialist physician.

Female respondents and younger respondents had a higher likelihood of reporting that their primary care physician was uninformed about the care they received from the specialist physician compared to male and older respondents. Respondents with a walk-in clinic in the previous 12 months also had a higher odds of reporting that their primary care physician was uninformed about the care they received from the specialist physician compared to non-walk-in users (aOR 1.40, 95% Cl 1.24–1.58). Respondents with higher general medical complexity had lower odds of reporting that their primary care physician was uninformed about their specialist care (aOR 0.91, 95% Cl 0.90–0.97). We found no significant association with the patient’s primary care model (Table 4).

## Discussion

We evaluated two patient-reported experience measures of information coordination between primary and specialist physicians in Ontario’s residents rostered to a primary care physician. Respondent’s experience of information coordination between primary care and specialist physicians was generally high. Respondents’ experience of information coordination generally did not differ across primary care models (except for those rostered in an enhanced FFS model). Walk-in clinic users reported lower information coordination with specialists. Older and complex respondents perceived that there was better information coordination between the primary care and specialist physicians.

By using a population-based multiyear patient-reported data, our study addresses the gap in previous studies (i.e., small sample size, focused on providers’ experience and disease-specific). Our findings align with the results of other surveys reporting high information coordination, especially among older age Canadians [43]. However, it appears that primary care physicians’ timely access to information from specialists remains a challenge [44]. Older respondents and those with complex health reported better information coordination, which may suggest that primary care and specialist physicians spend more time during visits with these patients [45] and the concentrated relationship primary care physician and specialists establish when caring for complex patients [46]. These findings may reflect years of investments and targeted government policies in improving health care for senior and complex patients [47, 48].

Enhanced FFS model of primary care and use of walk-in services appears to impact patients experience of information coordination between primary care and specialist physicians. Enhanced fee-for-service, fee-for-service and walk-in clinic models are financially incentivized to provide higher service volumes. Patients using walk-in clinics could often experience fragmented care given there is no formal relationship between the walk-in clinic physician and patients and information sharing could be challenging [49]. Thus, it is expected that respondents receiving care from these models would experience lower information coordination.

Information coordination could be supported with technological solutions that facilitate seamless information exchange between different levels of patients care achieving improved patients experience and quality of care. This could be supplemented by allowing patients to access their data, enabling them to actively participate in their care coordination and communicate with both their primary care provider and specialist physician. Also, our findings suggest that it is important to integrate walk-in clinics into the broader health care system to ensure continuity of patient information across different care providers. Patients with higher complexity scores may require additional support to feel supported, while also enabling seamless care transitions back to primary care.

### Limitations

By using multiyear patient-reported data, we have addressed many of the limitations of earlier studies. However, the survey design is limited to households with a valid health insurance card, community dwellers, and those with an active phone number. This could exclude refugees, people experiencing homelessness, and some Indigenous populations resulting in an underrepresentation of these populations. Although survey results were weighted to represent the overall population, we cannot rule out the potential for selection bias from the survey’s design and non-response bias due to a decline of the survey’s overall response rate over time. We included data before the COVID-19 pandemic, since the practice of primary care has shifted (i.e., expanded virtual care and e-consultations) in Ontario which could also impact patients’ perception of how information is coordinated between physicians [50, 51]. As with all survey studies, our findings are subject to recall bias (e.g., influenced by patients’ previous healthcare experiences) resulting in a possible risk of misclassification. Further, information coordination between primary care and specialist physicians could be influenced by factors such as patients’ expectations and health literacy, and physician’s characteristics such as physicians’ communication skills. Many of these factors were not measurable from the existing health administrative datasets or the survey; thus, they could not be included in this study. Our findings are generalizable to jurisdictions with universal primary care where the primary care physician are the gatekeepers for accessing publicly funded services from specialist physicians.

## Conclusion

Respondents reported high information coordination between their rostered primary care and specialist physicians. With exception to for respondents rostered to an enhanced FFS model of care, we did not find any difference across primary care models. Further research is needed to better understand physicians’ perspectives on the coordination of patient information and coordination of information with other sectors and providers (e.g., home care, acute care, pharmacists) given the shift in the role and function of primary care and increasing use of technology in delivering patient care.

## Supporting information

S1 Appendix Description of data sources. (DOCX)

S2 Appendix List of variables and their attributes. (DOCX)

S3 Appendix Unadjusted regression results for measuring rurality. (DOCX)

S4 Appendix STROBE checklist of items that should be included in reports of cross-sectional studies. (DOCX)

## Abbreviations

CAPE: Client Agency Program Enrolment
CCM: Comprehensive Care Model
CCO: Cancer Care Ontario
eFFS: Enhanced Fee-for-Service
FFS: Fee-for-Service
FHG: Family Health Group
FHN: Family Health Network
FHO: Family Health Organization
FHT: Family Health Team
HCES: Health Care Experience Survey
IPDB: ICES Physician Database
MLTC: Ministry of Long-Term Care
MOH: Ministry of Health
OHIP: Ontario Health Insurance Plan
OHIP Claims: Ontario Health Insurance Plan Claims Database
PCPOP: ICES Primary Care Population
RPDB: Registered Persons Database
RIO: Rurality Index of Ontario
