# Supplementary material for: Patients report high information coordination between rostered primary care physicians and specialists: A cross-sectional study
Source: PLoS One. 2024 Aug 22;19(8):e0307611. doi: 10.1371/journal.pone.0307611 (PMC11340953; doi:10.1371/journal.pone.0307611)
Supplement: S1 Appendix — (DOCX) [file pone.0307611.s001.docx]

**S1 Appendix A. Description of Data Sources.**

| **Name** | **Description** |
| --- | --- |
| Registered Persons Database (RPBD) | RPDB is a personal information bank that contains information on all Ontario residents registered for the Ontario Health Insurance Plan (OHIP) and Ontario Drug Benefit (ODB). |
| Health Care Experience Survey (HCES) | The HCES is a voluntary telephone survey given to an annual sample of 11,200 Ontarian residents age 16 years and older. Respondents are asked questions about their experiences with primary care, integration of specialists and primary care, the quality of their experiences, access to primary care for children living in the home, and socio-demographic information. The data is collected for the Ministry of Health/Ministry of Long-Term Care by Institute for Social Research is protected by law, and no identifying information is reported.  Before implementation, the survey was piloted between October 2012 and December 2012. The pilot involved ensuring respondents understood the questions, that the flow and order of questions were logical and easy to follow, that Computer Assisted Telephone Interview codes (skip patterns and conditional logic) were implemented correctly, and that all interviewer instructions were clear. Validation was completed at the Institute for Social Research to ensure that responses were consistent.  Respondent households receive a letter to notify them that they will be receiving a phone call from Institute of Social Research on behalf of Ontario’s Ministry of Health/Ministry of Long-Term Care for the HCES interview. Also, to further maximize the response rate, all telephone numbers receive up to 12 call attempts. More than 12 calls are made when there is a good reason to believe that additional calls will obtain interviews. In addition to making numerous call attempts and spreading these attempts over day, evening and weekend time slots, additional efforts are made to “convert” refusers in the last few weeks of each wave of data collection. |
| Client Agency Program Enrolment (CAPE) | CAPE contains a list of patients enrolled with a specific primary care enrolment physician model, including the patient's enrolment status (active or inactive). |
| ICES Primary Care Population (PCPOP) | PCPOP is a population-level dataset that includes all people in Ontario who are deemed alive and eligible at a given point in time. All indicators are as of the index date, with various look-back periods and an extensive list of primary care related indicators. |
| Ontario Health Insurance Plan Claims Database (OHIP) | OHIP contains most claims paid for by the Ontario Health Insurance Plan. The data cover all health care providers who can claim under OHIP (this includes physicians, groups, laboratories, and out-of-province providers). |
| ICES Physician Database (IPDB) | IPDB contains yearly information about all physicians in Ontario. Its potential uses include physician profiling, predicting physician behaviour, measuring physician supply, and many others. |
| CIHI Grouping Methodology | The Population Grouping Methodology (POP Grouper) using a case-mix classification case-mix classification to profile each person in the population using person level clinical information. It was developed by the Canadian Institute for Health Information (CIHI) to help meet the needs of Canada’s health care systems. |
| Note: The dataset from this study is held securely in coded form at ICES. Access could be granted to those who meet prespecified criteria for confidential access, available at *https://www.ices.on.ca/DAS/AHRQ (email: das@ices.on.ca).* | |
