# Supplementary material for: Patients report high information coordination between rostered primary care physicians and specialists: A cross-sectional study
Source: PLoS One. 2024 Aug 22;19(8):e0307611. doi: 10.1371/journal.pone.0307611 (PMC11340953; doi:10.1371/journal.pone.0307611)
Supplement: S2 Appendix — (DOCX) [file pone.0307611.s002.docx]

**S2 Appendix B. List of variables and their attributes.**

| **Variables** | **Data source** | **Definition/Codes** |
| --- | --- | --- |
| Sex (SEX) | RPDB | Sex in RPDB. Categorized as:  Female = 1  Male = 0 |
| Age (BYEAR) | RPDB | Age in RPDBD in years:  Number |
| Age – categorical (BYEAR) | RPDB | Age in RPDBD. Categorized as:  16-39 = 0  40-64 = 1  65-84 = 2  85+ = 3 |
| Rurality Index of Ontario Score (RIO) | RPDB | RIO score in RPDB. Categorized as:  Large urban (RIO 0) = 0  Medium urban (RIO 1-9) = 1  Small urban (RIO 10-39) = 2  Rural (RIO 40+) = 3 |
| Self-reported education (edu) | HCES | Survey question: What is the highest level of education you have completed to date? Categorized as:  High school (1, 2, 3) = 0  College or bachelor’s degree (4, 5, 6, 7) = 1  Graduate-professional degree (8, 9, 10) = 2  Missing (98, 99, .) = . |
| Self-reported financial situation (fin_sit) | HCES | Survey question: Which of the following words best describes your current financial situation: very comfortable, comfortable, tight, very tight, or poor?  Categorized as:  Very comfortable (1) = 1  Comfortable (2) = 2  Tight/very tight/poor (3, 4, 5) = 0  Don’t know or refused (8, 9) = 3 |
| Self-reported language most often spoken at home (lang_2) | HECE | Survey question: What language do you speak most often at home?  Categorized as:  English or French (1, 2) = 1  Other = 0 |
| Specialist physician informed about reasons for the visit (spec_4) | HCES | Survey question: When you last saw the specialist, did he/she have basic medical information from your provider about the reason for your visit?  Categorized as:  Yes (1) = 1  No (5) = 0  Still waiting (7) = 2  Don’t know or refused (8, 9) = 3 |
| Primary care physician informed about specialist care (spec_6) | HCES | Survey question: After you saw the specialist, did your provider seem informed and up-to-date about the care you got from the specialist?  Categorized as:  Yes = 1  No (5) =0  Didn’t see provider (7) = 2  Don’t know or refused (8, 9) = 3 |
| Self-reported time waited to see a specialist (spec_totdays) | HCES | Survey question: For your last appointment, how many days, weeks or months did you have to wait for an appointment? Reported as number of days. Categorized as:  2 weeks = 1  1 months = 2  2 months or more = 3 |
| Received conflicting information from primary care provider and specialist (spec_7) | HCES | Survey question: In the last 12 months, was there ever a time when you received conflicting information about your health care and needs from your provider and the specialist?  Categorized as:  Yes = 1  No = 0  Other = 2  Missing =. |
| Self-reported use of a walk-in clinic in the last 12 months (wi_1) | HCES | Survey question: Have you been to a walk-in clinic because you were sick or for a health-related problem in the last 12 months?  Categorized as:  Yes (1) = 1  No (5) =0  NA (7, 8, 9) = 2 |
| Primary care physician or clinic booked the appointment or coordinated care with the specialist (spec_3 - wave 6 and after) |  | Survey question: The last time you saw a specialist, did your provider or someone in their office book an appointment for you or coordinate the care you received from the specialist? Categorized as  Yes (1) = 1  No (5) = 0  NA/Refused/Don’t know (7, 8, 9) = 2 |
| Total visits to any specialist physicians over two years | OHIP | Count |
| Total visits to any primary care provider | OHIP | Count |
| Total visits to the rostered primary care physician over two years | OHIP | Count |
| Number of specialty types receiving care from | OHIP | Categorized as:  1 type = 1  2 types = 2  3 types or more = 3 |
| Complexity Score (Resource Intensity Weight – Concurrent) | Pop Grouper | Count |
| Types of primary care models (model_pcpop)  *Note: Assignment include formally and virtually rostered to the model over two years.* | PCPOP | From PROGTYPE2 in PCPOP within the six months of the interview date. Categorized as:  Virtually enrolled to a FFS = 0  Enhanced fee for service (Family Health Group or Comprehensive Care Model) = 1  Non-team Capitation (Family Health Network or Family Health Organization not associated with a Family Health Team) = 2  Team Capitation (Family Health Network or Family Health Organization associated with a Family Health Team) = 3  Other PEM models = 4 |
