# Supplementary material for: Patients report high information coordination between rostered primary care physicians and specialists: A cross-sectional study
Source: PLoS One. 2024 Aug 22;19(8):e0307611. doi: 10.1371/journal.pone.0307611 (PMC11340953; doi:10.1371/journal.pone.0307611)
Supplement: S3 Appendix — (DOCX) [file pone.0307611.s003.docx]

**S3 Appendix C. Unadjusted regression results.**

| **Respondents’ Characteristics** | **Unadjusted OR (CL)**  Specialist did not have basic medical information from the primary care physician about the reason for the visit | **Unadjusted OR (CL)**  Primary care physician seemed uninformed about the care received from the specialist physician |
| --- | --- | --- |
| Sex:  Male (ref)  Female | 1.25 (1.09 – 1.43) | 1.28 (1.14 – 1.43) |
| Age category  16-39 (ref)  40-64  65-84  85+ | 0.67 (0.58 – 0.78)  0.43 (0.36 – 0.52)  0.40 (0.24 – 0.65) | 0.55 (0.48 – 0.62)  0.25 (0.21 – 0.29)  0.24 (0.15 – 0.40) |
| Rurality:  Large urban (RIO score 0) (ref)  Medium urban (RIO score 1-9)  Small urban (RIO score 10-39)  Rural (RIO score 40+) | 0.91 (0.78 – 1.07)  0.80 (0.67 – 1.00)  0.70 (0.56 - 0.98) | 1.05 (0.92 – 1.20)  0.96 (0.83 – 1.11)  0.78 (0.64 – 0.99) |
| Self-reported education:  Graduate or professional degree (ref)  High school  College or bachelor’s degree | 0.76 (0.61 – 0.95)  1.12 (0.92 – 1.36) | 0.67 (0.55 – 0.81)  1.11 (0.95 – 1.30) |
| Self-reported financial situation:  Very comfortable (ref)  Comfortable  Tight/very tight/poor  Don’t know or refused | 1.14 (0.92 – 1.42)  0.97 (0.81 – 1.18)  1.16 (0.78 – 1.74) | 1.41 (1.17 – 1.69)  1.06 (0.89 – 1.24)  0.89 (0.58 – 1.37) |
| Language most often spoken at home:  English or French (ref)  Other than English or French | 1.89 (1.72 – 2.12) | 1.10 (1.01 – 1.28) |
| Self-reported waited to see a specialist:  2 weeks (ref)  3-8 weeks  More than 8 weeks | 0.91 (0.77 – 1.07)  1.11 (0.93 – 1.31) | 1.23 (1.06 – 1.42)  1.64 (1.41 – 1.91) |
| Number of specialty types receiving care from:  1 type (ref)  2 types  3 types or more | 0.66 (0.52 – 0.84)  0.57 (0.48 – 0.68) | 0.83 (0.68 – 1.01)  0.57 (0.48 – 0.67) |
| Types of primary care models:  Team Capitation (ref)  Solo FFS  Enhanced FFS  Non-team Capitation  Other PEM models | 1.29 (0.92 – 1.80)  1.28 (1.09 – 1.52)  1.17 (1.01 – 1.38)  0.75 (0.36 – 1.57) | 1.17 (0.87 – 1.57)  1.07 (0.93 – 1.23)  0.98 (0.85 – 1.13)  0.94 (0.59 – 1.38) |
| Self-reported use of a walk-in clinic in the last 12 months:  No (ref)  Yes  I don’t know/Refused | 1.62 (1.41 – 1.86)  2.95 (1.46 – 5.96) | 1.74 (1.55 – 1.95)  2.17 (0.12 – 1.43) |
| Complexity score based on CIHI Pop Grouper (1 unit increase) | 0.95 (0.92 – 0.98) | 0.85 (0.82 – 0.88) |
| Total visits to the rostered primary care physician over two years (1 visit increase) | 0.98 (0.97 – 0.99) | 0.97 (0.95 – 0.98) |
| Total visits to any specialist physicians over two years (1 visit increase) | 0.99 (0.98 – 0.99) | 0.98 (0.97 – 0.99) |
